# The Complexity of Health Service Integration: A Review of Reviews

**DOI:** 10.3389/fpubh.2016.00223

**Published:** 2016-10-17

**Authors:** Marion Heyeres, Janya McCalman, Komla Tsey, Irina Kinchin

**Affiliations:** ^1^The Cairns Institute, James Cook University, Cairns, QLD, Australia; ^2^School of Human Health and Social Sciences, Central Queensland University, Cairns, QLD, Australia

**Keywords:** integration, health service, health-care system, collaboration, governance

## Abstract

**Background:**

The aim of health service integration is to provide a sustainable and integrated health system that better meets the needs of the end user. Yet, definitions of health service integration, methods for integrating health services, and expected outcomes are varied. This review was commissioned by Queensland Health, the government department responsible for health service delivery in Queensland, Australia, to inform efforts to integrate their mental health services. This review reports on the characteristics, reported outcomes, and design quality of studies included in systematic reviews of health service integration research.

**Method:**

The review was developed by systematically searching nine electronic databases to find peer-reviewed Australian and international systematic reviews with a focus on health service integration. Reviews were included if they were in the English language and published between 2000 and 2015. A standardized assessment tool was used to analyze the study design quality of included reviews. Data relating to the integration types, methods, and reported outcomes of integration were synthesized.

**Results:**

Seventeen publications met the inclusion criteria. Eleven (65%) reviews were published during the past 5 years, which may indicate a trend for increased awareness of the need for service integration. The majority of reviews were published by researchers in the UK (8/47%), USA (3/18%), and Australia (3/18%). Included reviews focused on a variety of integration types, including integrated care pathways, governance models, integration of interventions, collaborative/integrated care models, and integration of different types of health care. Most (53%) of the reviews reported on the cost-effectiveness of service integration, e.g., positive results, no effect, or inconclusive. Only one of the reviews reported on the importance of consumer involvement. The overall design of 70% of the reviews was high, 18% medium, and 12% low.

**Conclusion:**

There is no “one size fits all” approach to health service integration. Instead, this literature review highlighted the complexity of service integration, which in most primary studies involved a range of strategies. Rigorous assessments of cost-effectiveness and reporting on consumer involvement are required in future research.

## Introduction

Service integration is seen as a useful approach for building a more efficient health-care system that takes a patient-centered focus and results in improved health outcomes. In 2007, WHO Director General [(1), p.1] stated:

“We need a comprehensive, integrated approach to service delivery. We need to fight fragmentation.”

Globally, health services face resource constraints, with a particular issue of human resource shortages in low-income countries. A variety of approaches to service integration can be witnessed throughout the world. In low-income countries, there is a noticeable focus on increasing health coverage through primary health care as well as promoting community participation (2). In middle-income countries, perhaps the most common strategies to applying the principles of health services integration are the strengthening of primary and community care practices; the adoption of new laws and regulations in the prevention and control of chronic disease; and the development of community-based interventions that engage and empower people to adopt healthier lifestyles, support better disease management, and enable community rehabilitation and independent living. In high-income countries, aging populations and the growing burden of long-term chronic illness places significant strain on health care systems. In response, many national governments have instituted structural and financial reforms to promote integrated care and, specifically, encourage intersectoral action with social services such as housing, employment, family welfare, and disability support programs (2). In Australia, Health Ministers agree that collaboration across jurisdictions, including joint initiatives and shared learning, can help all parties to achieve a sustainable, integrated health system that better meets the needs of Australians (3). As an example, the National Primary Health Care Strategy requests the Commonwealth and State governments to identify possible models of multidisciplinary team care coordination and/or case management to keep people healthy and reduce avoidable hospitalizations (4).

As a result of the increasing awareness for the need of service integration, hundreds of studies have been published worldwide. The best way to achieve an overview of all the work available is to conduct a systematic review. Systematic reviews involve a rigorous scientific approach to an existing body of research evidence. Procedures are explicitly defined in advance, in order to ensure that the exercise is transparent and can be replicated. This practice is also designed to minimize bias (5). Original research is identified, eligible studies are critically appraised, and results summarized and synthesized (6). To date, systematic reviews of international literature relating to service integration in health have focused mainly on four key types of integration: (1) integration around specific health issues, e.g., HIV/AIDS or depression care; (2) within and across aspects of health systems, e.g., the integration of particular services or interventions; (3) integrated care pathways within primary health care, e.g., stroke care, care of chronically ill; and (4) collaboration across services, e.g., maternity, child, and family care. The majority of literature available pertaining to service integration focusses on issues in developed countries, with a smaller share of the pie on issues in developing countries.

However, decision makers are now faced with large numbers of systematic reviews and syntheses available on any given topic, and available systematic reviews are likely to differ in quality and scope. Reviews of systematic reviews are a logical and appropriate next step to provide comparison and contrast of the findings of separate systematic reviews, providing decision makers with the evidence they need in a single manuscript (7). This review aims to assist decision makers and planners to better understand, develop, and implement integrative approaches through time-saving access to the latest available evidence across the health service integration knowledge base.

### Objectives

The objective of this review was to review existing systematic literature reviews on health service integration to determine how studies report the characteristics of included studies, service integration types, methods, and outcomes. The research questions were determined as follows:

1. What are the characteristics of studies in the included reviews?
2. What are the reported study outcomes of included reviews?
3. What is the design quality of the included reviews?

#### Definition

As “health services integration” has been variously defined, for the purpose of this review, we chose to combine two concepts: (a) that service integration is a continuum rather than two extremes of integrated/not integrated, and (b) the benefits of service integration are focused on the customer. The following definition was, therefore, used for the purpose of this review: “a variety of managerial and operational changes to health systems that bring together inputs, delivery, management, and organizations of particular service functions, in order to provide clients with a continuum of preventative and curative services, according to their needs over time and across different levels of the health system” (8, 9).

## Methods

### Search Strategy for the Identification of Studies

Nine electronic databases were searched including JBI, Prospero, The Cochrane Library, The Campbell Library, Medline, Cinahl, Scopus, PsychArticles, and PsychInfo. The internet search engine Google Scholar was also searched. Included studies focused on service integration in health, and the following term combinations were searched in the title and abstract: (systematic review) and effective* (service integration) and (primary care); (literature review) and (cost-effective models of service integration) and (primary health); (“service* integration model*” or “service* integration” or “intersectional collaboration” or “interagency collaboration” or “partners in care” or “structural model*”) and (youth or child* or adoles*) and health; (“service* integration model*” or “service* integration” or “intersectional collaboration” or “interagency collaboration” or “partners in care” or “structural model*”) and (youth or child* or adoles*) and (primary health) and effective and integrated; (“health care” or “mental health care”) and (model*) or “case management” or “delivery of health care” or integrated and (youth or child* or adoles*). Separate searches were performed for each database using database specific subject headings and keywords. The citation trail from the Primary health care position statement: a scoping of the evidence (10) was followed and hand searched for targeted journals.

### Inclusion and Exclusion Criteria

The following inclusion criteria were applied to reviews: identified as a systematic review and/or followed an explicit and systematic approach, based on a clearly formulated question, identified relevant studies, appraised their quality, and summarized the evidence by use of an explicit methodology (11). International publications from 2000 to 2015 were included in the review. Only publications in English were included. Gray literature was not searched as the focus of the review was on peer-reviewed publications only. Literature outside the chosen time period from 2000 to 2015 was not included.

### Review Process

**Figure 1:**
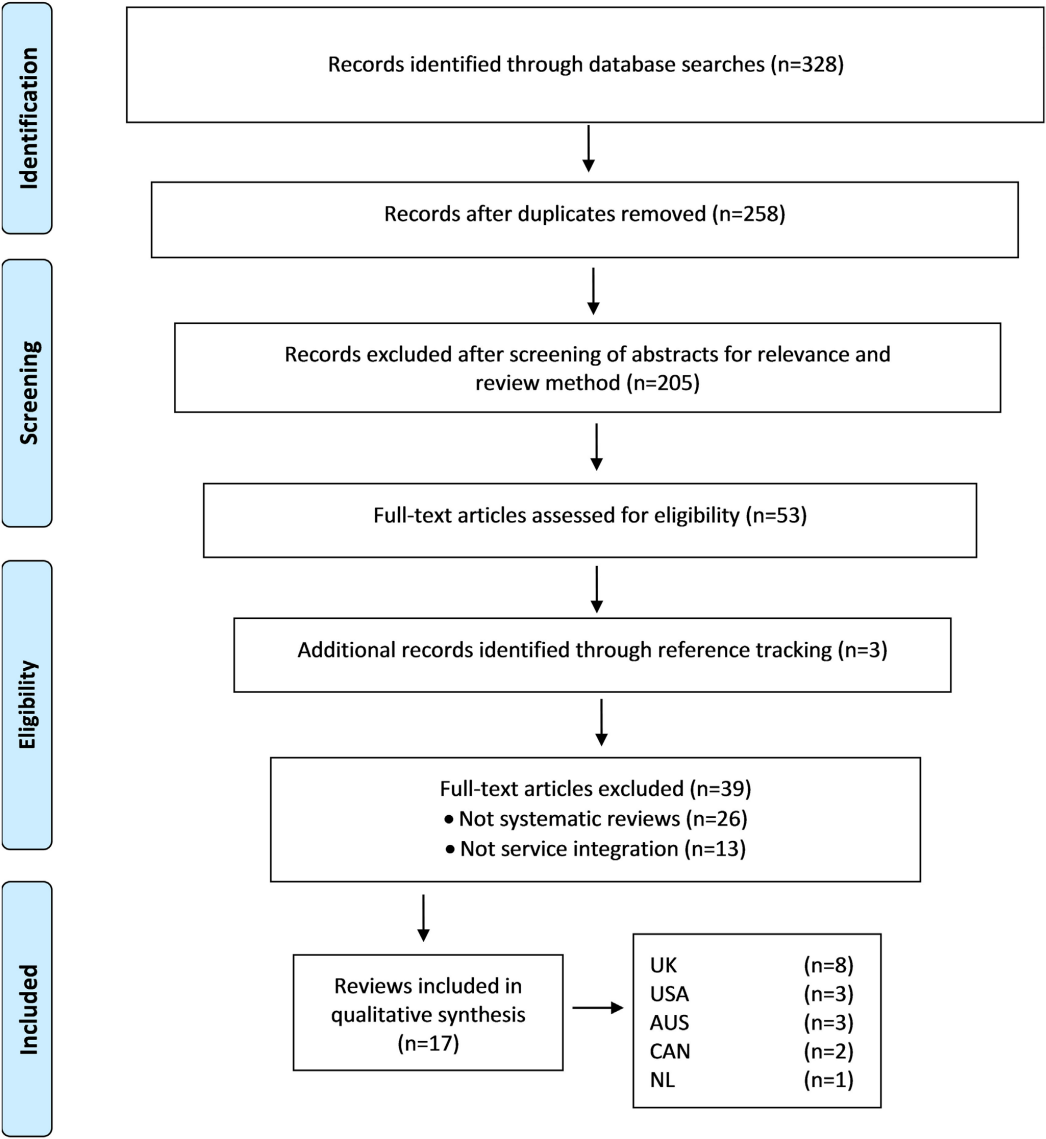
**Flowchart representing the selection process for publications included in the review**. From Moher et al. (13). For more information, visit www.prisma-statement.org.

The process used to identify and classify publications is consistent with the Cochrane methods for systematic searches (12). Search results were imported into bibliographic citation management software (Endnote X7). We retrieved 328 potentially relevant publications through database searches and after duplicates were removed, 258 records remained. In the next step, two reviewers screened abstracts for relevance and type of review method, which reduced the selection to 53 potentially suitable publications. At this stage, an additional three publications were identified by reference tracking. Where necessary, the full text was obtained to determine the eligibility of the publication for inclusion. Publications that were not a systematic review and had no focus on service integration were removed, which resulted in 17 systematic reviews that were included in this study. Results of the search strategy were recorded in an Excel spread sheet against the database name and search strings used. Figure 1 provides a Preferred Reporting Items for Systematic Reviews and Meta-Analyses (PRISMA) study flow diagram to illustrate the search strategy employed.

### Classification of Reviews

Included reviews were categorized by the first author and publication year, geographic location (country), and quality of review design. Data relating to service integration were analyzed by integration type, reported outcomes and methods, and focus on consumer involvement (Table 1). Integration type was categorized according to vertical integration, e.g., clear pathways, smooth handovers between services, and coordinated plans for forward movement; or horizontal integration, e.g., networks and partnerships between services, interdisciplinary teams, and consumer engagement (14). Consumer involvement was categorized according to whether the reviews reported on consumers being involved in the planning, implementation, and/or evaluation of an intervention.

**Table 1:** **Summary of characteristics of all included reviews**.

Reference	Country	Integration type/methods	Reported outcomes on	Consumer involvement
Allen and Rixson (15)	UK	Integrated care pathways	Cost-effectiveness	No
Armitage et al. (16)	CAN	Model for health systems integration	Cost-effectiveness	No
Atun et al. (17)	UK	Integration of population, health, and nutrition	Cost-effectiveness	No
		interventions into mainstream health systems		
Atun et al. (18)	UK	Integration of population health and nutrition interventions into mainstream health systems in developing countries	Cost-effectiveness	No
Bower et al. (19)	UK	Collaborative care intervention in primary care	Consumer related	No
			Staff related	
Bradford et al. (20)	USA	Care models for individuals with serious mental illness	Consumer related	No
			Structure/governance related	
Davies et al. (21)	UK	Integration of health care services into care homes	Cost-effectiveness	No
Lindegren et al. (22)	USA	Integration of HIV/AIDS services into MNCHN-FP (maternal, neonatal, child health, nutrition, and family planning) services	Consumer related	No
			Structure/governance related	
Nicholson et al. (23)	AU	Governance model for integrated primary/secondary care in health	Cost-effectiveness	Yes
			Consumer related	
			Staff related	
			Structure/governance related	
Ouwens et al. (24)	NL	Integrated care programs for chronically ill patients	Cost-effectiveness	No
			Consumer related	
			Staff related	
			Structure/governance related	
Suter et al. (25)	CAN	Model for health systems integration	Consumer related	No
			Staff related	
			Structure/governance related	
Suthar et al. (26)	UK	Service integration and decentralization to improve antiretroviral therapy uptake and effectiveness	Staff related	No
			Structure/governance related	
Sweeney et al (27)	UK	Integration of HIV/AIDS services with other health services	Cost-effectiveness	No
			Structure/governance related	
Tan et al. (28)	AU	Integration of pharmacist services with general practice clinics	Consumer related	No
			Structure/governance related	
Tieman et al. (29)	AU	Multidisciplinary teams for the care of chronically ill or frail aged	Cost-effectiveness	No
			Consumer related	
			Structure/governance related	
Tudor Car et al. (30)	UK	Integration of perinatal interventions with other health care services in developing countries	Consumer related	No
Woltmann et al. (31)	USA	Collaborative chronic care models for mental health conditions	Cost-effectiveness	No

### Data Synthesis

An explorative approach and a narrative summary of findings were taken to interrogate the data relating to (a) integration type, (b) methods, and (c) reported outcomes. Synthesis of these data resulted in the presentation of key themes.

### Quality of Review Design

To determine the quality of the review designs, we followed the recommendations of West et al. (32) on “systems for rating the quality of systematic reviews.” The seven key domains suggested to be of importance for a high quality review were: study question/aim, search strategy, inclusion and exclusion criteria, data abstraction, study quality and validity, data synthesis and analysis, and funding and sponsorship (Table 2).

**Table 2:** **Reported outcomes on cost-effectiveness; consumer, staff, and structure/governance**.

Reference	Cost-effectiveness	Consumer related	Staff related	Structure/governance related
Allen and Rixson (15)	✓			
Armitage et al. (16)	✓			
Atun et al. (17)	✓			
Atun et al. (18)	✓			
Bower et al. (19)		✓	✓	
Bradford et al. (20)		✓		✓
Davies et al (21)	✓			
Lindegren et al. (22)		✓		✓
Nicholson et al. (23)	✓	✓	✓	✓
Ouwens et al. (24)	✓	✓	✓	✓
Suter et al. (25)		✓	✓	✓
Suthar et al. (26)			✓	✓
Sweeney et al. (27)	✓			✓
Tan et al. (28)		✓		✓
Tieman et al. (29)	✓	✓		✓
Tudor Car et al. (30)		✓		
Woltmann et al. (31)	✓			
Percentage of all included reviews	59%	53%	29%	53%

## Results

### Characteristics of Reviews

Seventeen reviews met the criteria for inclusion. A summary of the characteristics of included reviews is provided in Table 1.

#### Publication Date

Over half of included studies (59%) were published during the past 5 years, which may indicate a trend for increased awareness of the need for service integration.

#### Geographic Location

The majority of reviews were published by researchers in the UK (8/47%), USA (3/18%), and Australia (3/18%), only two (11%) were from Canada and one (6%) from the Netherlands.

#### Integration Type/Methods

Based on what was reported, we took a grounded approach in categorizing included reviews in this study into five different types: (1) integrated care pathways, (2) governance models, (3) integration of interventions, (4) collaborative/integrated care models, and (5) health care service integration. One review (6%) focused on integrated care pathways, which map out a patient’s journey and provide coordination of services for users. Allen (15) concisely described them as “Having the right people, doing the right things, in the right order, at the right time, in the right place, with the right outcome”. Three reviews (18%) reported on governance models for health systems integration, which provide guidance on the planning and implementation of service integration (16, 23, 25). The focus of three reviews (18%) was on integration of particular interventions, such as population health and nutrition interventions into mainstream health systems, or peri-natal intervention with other health care services in developing countries (17, 18, 30). The most common types of service integration reported were collaborative/integrated care models and the integration of various types of health services. Five reviews (29%) focused on collaborative care and integrated care models within primary care, e.g., for patients with mental illness, chronically ill or frail aged (19, 20, 24, 29, 31). The remaining five reviews (29%) focused on the integration of different types of health care services, such as HIV/AIDS services and antiretroviral therapy into maternal, neonatal, child-health, nutrition, and family planning services; the integration of health care services into care homes, or pharmacist services into general practice clinics (21, 22, 26–28).

#### Consumer Involvement

Given the documented importance of consumer involvement in the planning, implementation and evaluation of interventions, only one of the included reviews reported on it. Nicholson et al. (23) found the consumer’s voice to be a requirement to support joint planning, a key element in developing integrated care across the primary and secondary care continuum.

### Reported Outcomes

The reported outcomes are categorized into four themes. Ten (59%) out of all included reviews reported on cost-effectiveness, nine (53%) on consumer related outcomes, five (29%) on staff related outcomes, and nine (53%) on structure/governance related outcomes.

#### Cost-effectiveness

Of the 17 included reviews, more than half (59%) reported on the cost-effectiveness of service integration. Positive results were reported by Sweeney et al. (27) on the reductions in facility costs due to joint utilization. Integrated HIV services were found to be cost-effective compared with “do-nothing” alternatives. Atun (17, 18) found that the implementation of integrated management of childhood illnesses was not associated with higher costs than routine care, but led to significant improvement in case management. There was no effect on the total health care costs reported by Woltmann et al. (31) between collaborative chronic care and comparison models. Armitage (16) reported conflicting findings, e.g., in some cases, financial performance increased, or cost per patient visit reduced while other studies found no improvement. Two reviews, Allen and Rixson (15) and Davies et al. (21) reported insufficient available information to evaluate the costs associated with the management of integrated care pathways, or the work between care homes and primary health care professionals. Ouwens et al. (24) concluded that to compare and better understand the cost-effectiveness of integrated care programs, consistent definitions must be used and component interventions must be well described. Overall, despite varying results, having the ability to integrate clinical and financial information, across health and social care, seemed important for monitoring cost-effectiveness (23).

#### Consumer-Related Outcomes

Nine (53%) of the included reviews reported on consumer-related outcomes. Consumers in the UK were presented more and more with opportunities for input on various levels, and hence distinguished between consumer input in the “delivery of services” and into “partnerships for better health” (23). Partnerships reviews reported on consumer inputs into patient/carer experiences and satisfaction to improve performance, as well as community forums and large public meetings that present health plans and seek views about what people wanted from the new health care system (23, 25). Joint planning offered opportunities for health-care users and providers to come together and use information to arrive at a shared vision of optimal health care (23). In agreement with Nicholson, Suter et al. (25) pointed out that integrated health systems should be easy for patients to navigate and stressed the importance of involving consumers and representatives of the served communities. However, as it may be challenging for large integrated systems to retain a patient focus, smaller systems were seen as having better chances at doing so (25). Consumer self-management support and education were reported by Ouwens et al. (24) as some of the most common components of integrated care programs. Provision of opportunities for consumers to actively engage in their treatment process was reported by Nicholson et al. (23) and included internet-based lab results display and results trending over time, clinical reminders, self-scheduling, secure e-mail with providers, prescription refills, and educational content. Bower et al. (19) reported collaborative care to be no significant predictor of the effect on anti-depressant use.

#### Staff-Related Outcomes

Five out of 17 reviews (29%) reported on components relating to staff. These studies recommended that staff members receive adequate training and mentoring; management was to be fully committed and in support of integration; leaders should have a clear vision of the importance of integrated care; continuous professional development was required to support joint working; health worker training and supervision; patient care teams should be multidisciplinary; and incentives provided (19, 23–26).

#### Structural/Governance-Related Outcomes

Varied structural/governance level components for successful integration were reported in nine (53%) reviews. They included joint planning and management, resource mobilization and sharing; integrated information and communication technology; sound financial management for implementation and maintenance; multidisciplinary clinical pathways and feedback and reminders; innovation; a culture of quality improvement; structured clinical follow-up and case management; evidence-based clinical care guidelines and protocols; geographic coverage and rostering to maximize accessibility and minimize duplication; specialized clinics or centers; primary care physician integration; strong governance structure; and strong data collection systems and performance management (20, 22–29).

### Quality of Review Design

As shown in Table 3, the overall quality of review design of twelve included publications (70%) was rated as high (17–23, 25, 27–30), three (18%) as medium (15, 26, 31), and two as low (12%) (16, 24). Fifteen of the 17 reviews (88%) clearly stated their aim or made their research question explicit (15, 17, 19–31). Thirteen (76%) provided a clear search strategy in the form of a diagram (15, 17–19, 21–23, 25–30). Fifteen reviews (88%) reported on the reasons for including or excluding certain publications (15, 17–23, 25–31). Fifteen reviews (88%) presented essential characteristics of their data in the form of a table (16–28, 30, 31). Thirteen (76%) reviews explained in detail the analysis of the methodological quality of their included publications (15, 17–23, 25, 27–30). Fifteen reviews (88%) provided a synthesis of their data rather than a mere description of studies (17–31). Only eight reviews (47%) acknowledged a funding body or sponsor (20–22, 25, 27, 29–31). The overall strength of evidence of included reviews in this review resulted from the average scores of the seven key categories, as described above.

**Table 3:** **Seven key domains that determined the quality of review design**.

Reference	Study question/aim	Search strategy	Inclusion and exclusion criteria	Data abstraction	Study quality and validity	Data synthesis and analysis	Funding or sponsorship	Overall quality
Allen and Rixson (15)	✓	✓	✓	No	✓	No	No	Medium
Armitage et al. (16)	No	No	No	✓	No	No	No	Low
Atun et al. (17)	✓	✓	✓	✓	✓	✓	No	High
Atun et al. (18)	No	✓	✓	✓	✓	✓	No	High
Bower et al. (19)	✓	✓	✓	✓	✓	✓	No	High
Bradford et al. (20)	✓	No	✓	✓	✓	✓	✓	High
Davies et al. (21)	✓	✓	✓	✓	✓	✓	✓	High
Lindegren et al. (22)	✓	✓	✓	✓	✓	✓	✓	High
Nicholson et al. (23)	✓	✓	✓	✓	✓	✓	No	High
Ouwens et al. (24)	✓	No	No	✓	No	✓	No	Low
Suter et al. (25)	✓	✓	✓	✓	✓	✓	✓	High
Suthar et al. (26)	✓	✓	✓	✓	No	✓	No	Medium
Sweeney et al. (27)	✓	✓	✓	✓	✓	✓	✓	High
Tan et al. (28)	✓	✓	✓	✓	✓	✓	No	High
Tieman et al. (29)	✓	✓	✓	No	✓	✓	✓	High
Tudor et al. (30)	✓	✓	✓	✓	✓	✓	✓	High
Woltmann et al. (31)	✓	No	✓	✓	No	✓	✓	Medium

## Limitations

Systematic and rigorous methods were used to provide a summary of the current literature on service integration in health research. Only peer-reviewed papers published in English were reviewed, hence potentially relevant articles and information from primary research and gray literature published in other languages may have been missed. Most of the results presented in the included reviews did not allow for comparison across studies. However, given the volume of literature on service integration, a search of systematic reviews across the health care system is an efficient way of providing a summary of the current evidence.

## Discussion and Conclusion

This study set out to review the current available research data from systematic literature reviews on health service integration to assist decision makers in their decision making process for developing better health policy. A substantial amount of national and international literature is available on the importance of service integration in health that describes different models and strategies. However, this literature search highlighted service integration is complex and varied. There are a range of strategies, e.g., integrated care pathways, governance models, integration of interventions, collaborative/integrated care models, and health-care service integration. We conclude that there is no “one size fits all” approach. However, the key ingredient identified for implementing appropriate models, processes, strategies, and structures was to be clear about the purpose of integration and understand what needs to be integrated (16).

About half of the included reviews reported on the cost-effectiveness of service integration in terms of positive outcomes, no effects, and inconclusive results. Having the ability to integrate clinical and financial information across health and social care was seen to be important for monitoring cost-effectiveness (23). However, only one study (27) was dedicated to providing a cost evaluation, while several others reported on the effectiveness of the integration in terms of service and medicinal uptake, and service access. This lack of robust economic evaluations seems surprising, considering the overarching emphasis in the literature on the potential of service integration to improve the effectiveness of health services. We conclude that more research needs to be done in this area to support decision makers with high quality economic data. Nonetheless, considering that a high 70% of the included publications were rated of high quality review design (17–23, 25, 27–30), we are suggesting decision makers may feel confident that the available, evidence-informed data have been achieved by the application of rigorous research methodologies. This is a very encouraging result.

Across the general health literature, there is an overall notion that involving consumers in the planning and implementation of service integration may increase the likelihood of services uptake, and also improve outcomes for them. However, only one of the included publications (23) reported on active consumer involvement. This suggests that more emphasis must be given on involving consumers, if we are to create a truly patient-centered health-care system.

## Author Contributions

MH conceived the research questions and prepared the research protocol in conjunction with KT. MH designed the study, conducted the literature searches, and analyses. MH and KT refined the refined the research question in the first draft. JM and IK contributed to the study design, participated in the literature review, helped prepare the first draft and revisions. All authors read and approved the final version of the manuscript for submission.

## Conflict of Interest Statement

The authors declare that the research was conducted in the absence of any commercial or financial relationships that could be construed as a potential conflict of interest.
